# Public health research using cell phone derived mobility data in sub-Saharan Africa: Ethical issues

**DOI:** 10.17159/sajs.2023/14777

**Published:** 2023-05-30

**Authors:** Stuart Rennie, Caesar Atuire, Tiwonge Mtande, Walter Jaoko, Sergio Litewka, Eric Juengst, Keymanthri Moodley

**Affiliations:** 1 Department of Social Medicine, University of North Carolina at Chapel Hill, Chapel Hill, North Carolina, USA; 2 UNC Center for Bioethics, University of North Carolina at Chapel Hill, Chapel Hill, North Carolina, USA; 3 Department of Philosophy and Classics, University of Ghana, Accra, Ghana; 4 Centre for Medical Ethics and Law, Department of Medicine, Faculty of Medicine and Health Sciences, Stellenbosch University, Cape Town, South Africa; 5 KAVI-Institute of Clinical Research, University of Nairobi, Nairobi, Kenya; 6 Institute for Bioethics and Health Policy, Miller School of Medicine, University of Miami, Miami, Florida, USA

**Keywords:** ethics, mobility data, public health, community engagement, surveillance

## Abstract

The movements of humans have a significant impact on population health. While studies of such movements are as old as public health itself, the COVID-19 pandemic has raised the profile of mobility research using digital technologies to track transmission routes and calculate the effects of health policies, such as lockdowns. In sub-Saharan Africa, the high prevalence of cell phone and smartphone use is a source of potentially valuable mobility data for public health purposes. Researchers can access call data records, passively collected in real time from millions of clients by cell phone companies, and associate these records with other data sets to generate insights, make predictions or draw possible policy implications. The use of mobility data from this source could have a range of significant benefits for society, from better control of infectious diseases, improved city planning, more efficient transportation systems and the optimisation of health resources. We discuss key ethical issues raised by public health studies using mobility data from cell phones in sub-Saharan Africa and identify six key ethical challenge areas: autonomy, including consent and individual or group privacy; bias and representativeness; community awareness, engagement and trust; function creep and accountability; stakeholder relationships and power dynamics; and the translation of mobility analyses into health policy. We emphasise the ethical importance of narrowing knowledge gaps between researchers, policymakers and the general public. Given that individuals do not really provide valid consent for the research use of phone data tracking their movements, community understanding and input will be crucial to the maintenance of public trust.

## Introduction

The use of big data for public health promotion, clinical care improvement and health system strengthening is increasingly globalised. Until the recent past, the practice of collecting, merging, storing and using large data sets for these purposes was mostly limited to high-income countries. This is no longer the case. Persistent health challenges and the rising integration of digital technologies in the daily lives of people in sub-Saharan Africa (SSA) have engendered increased interest in big data initiatives throughout the region. Traditional health statistics, such as those gathered by government agencies through demographical and health surveys, are unlikely to be as comprehensive as vast volumes of health-related data gathered in real time from a diversity of born digital sources by both public agencies and private companies.

The development and adoption of Internet telephony has been of particular interest to those who have worked for many years to improve health in SSA. In the colonial and post-colonial periods, the use of landlines in Africa was limited to government offices, businesses in major cities and socio-economic elites. Over the past two decades, the rarity of landlines has been eclipsed by the ubiquitous use of cell phones.^1^ According to the Global System for Mobile Communications report, *The Mobile Economy Sub-Saharan Africa 2022*, smartphones are being rapidly adopted: on average accounting for 49% of connections in 2022, smartphones are predicted to account for 61% of connections by 2025. However, this means that nearly 40% of people in SSA use basic cell phones or have no mobile phone access at all.^2^ The widespread use of cell phones, in turn, has sparked the rise of mobile health – or mHealth – initiatives and research studies that seek to improve health and healthcare services by enhancing communication between patients and healthcare providers, study participants and researchers, and citizens and public health professionals. Many mHealth applications (apps) for public health purposes have been developed, implemented and evaluated in low- and middle-income countries (LMICs).^3–5^ Tomlinson et al.^6^ reported on the use of cell phones by community health workers in South Africa to conduct electronic household surveys and questionnaires. Cell phone surveys could be a cost-effective approach to gather data about non-communicable diseases in LMICs.^7^ Similarly, Brinkel et al.^8^ describe the use of cell phones in public health surveillance, i.e. where health workers gather patient (and sometimes Global Positioning System [GPS]) data on cell phones and send the information to the server of a local cell phone service provider, upon which the data are forwarded to district health offices and research institutes.

This paper addresses ethical issues that arise with one particularly powerful form of such big data research: the use of cell phone data to track human mobility patterns in efforts to improve public health in SSA. How people move affects population health, most obviously in the case of infectious disease epidemics, such as the West African Ebola outbreak^9^ and the COVID-19 pandemic^10–12^. This research utilises call data records (CDRs) that cell phone companies passively collect in real time from millions of clients. CDRs list the cellular base station or tower and the code of the subscriber identification module (SIM) card involved in each call or text. If these data are available, in conjunction with a map of the relevant towers, the location of each call or text can be identified and, from this, an individual’s movement between calls can be derived.^13^ The resulting data, which often involves hundreds of thousands of trajectories, can be associated with other data sets to generate insights, make predictions or draw possible policy implications. For example, Gibbs et al.^14^ used data from CDRs in Ghana to identify the relationship between reductions in human mobility and decreases in the real-time reproduction number (R_t_) of COVID-19 during the early stages of the pandemic. In addition to CDRs, other types of location data are collected through cell phone apps or the Bluetooth functionality on smartphones. Although this paper focuses on data from CDRs, the ethics discussion is also applicable to other types of cell phone location data.

Cell phone companies can share data from CDRs with third parties (including researchers) in different ways. De Montjoye et al.^15^ offer four ‘privacy-conscientious’ models of CDR data sharing, where anonymisation and the spatial or temporal aggregation of shared data are central. According to a limited release model, the telecommunications company technically transforms a cell phone data set so as to make the reidentification of individuals very difficult. According to a precomputed indicators and synthetic data model, third parties are not given transformed data, but receive information derived from a cell phone data set (such as number of calls) or synthetic data that convey the predefined statistical properties of the original data set. According to a remote access model, the data are not released, but stay under the control of the telecommunications company (or authorised authority) and can be conditionally accessed by third parties remotely. Finally, in a question-and-answer model, the data set remains under the control of the telecommunications company and is accessed by third parties through a question-and-answer system: third parties ask specific and standardised questions about a data set, and the telecommunications company provides answers that have been vetted by a security and auditing system. The limited release model is closest to traditional data sharing. Given that it requires fewer technical and human resources than the alternatives, this model is likely the predominant CDR data-sharing model in SSA.

The research use of cell phone derived mobility data is likely to lead to a variety of public health benefits in SSA, even if it is difficult to identify or quantify them at this early stage. Drawing lessons from the COVID-19 pandemic response, Oliver et al.^16^ describe four areas in which mobility data can be beneficial for epidemic control: understanding the dynamic environment of an epidemic; tackling cause-and-effect questions by identifying the key mechanisms and consequences of epidemic containment; predicting the likelihood of future outcomes; and developing impact assessments to determine how various interventions impact epidemic spread. To optimise these and other social benefits, it will be important for researchers and data managers to anticipate the ethical and social challenges that may arise along the way. Our focus here is on key ethical challenges raised by the use of these data in the SSA context, particularly considering that there are no formal ethics guidelines specific to mobility science, and such research is likely to be unfamiliar to most research ethics committees in the region. This review makes use of diverse literature: mobility research related to development, migration and humanitarian crises; anthropological research on cell phone use in Africa; current debates about mobility justice; and mobility data related to health promotion in LMICs, particularly in Africa. The core themes are also derived from the involvement of the authors in the Data Science for Health Discovery and Innovation in Africa (DS-I Africa) initiative and from relevant discussions within bioethics circles in SSA. They are not additionally embedded within dominant bioethics frameworks, which originate from high-income countries and whose universality has been placed in question in current discourses surrounding the decolonisation of bioethics.^17^ Based on this review, we address six challenge areas: autonomy, including consent and individual or group privacy; bias and representativeness; community awareness, engagement and trust; function creep and accountability; stakeholder relationships and power dynamics; and the translation of mobility analyses into health policy.

### Autonomy

Respect for the autonomy of research participants is a core value in research, usually represented in terms of obtaining their free and informed consent, protecting participants’ privacy, and ensuring their ability to withdraw at any time. However, each of these standard methods of respecting the rights and interests of human data sources faces particular challenges in the research use of cell phone derived mobility data in the SSA context.

In their privacy policies and the terms of their service agreements, cell phone companies often disclose that client data may be shared with third parties. Mobility researchers are among these parties, and it could be argued that a client who agreed to the cell phone companies’ privacy policies and terms of service therefore consented to the collection, sharing and use of their call records. Although this might be legally adequate, whether this is an ethically valid form of consent has long been debated.^18^ Extensive, densely written and sometimes buried policies, which require the client to agree in full or forgo the service, are often poorly read or understood, drawing doubt on whether consent is voluntary and informed.^19^ This is even more relevant in the African rural context where users of cell phones may have less information about the technological infrastructure underlying cell phone use and how data are collected. Cell phone operators generally do not offer consent forms in local languages, making it difficult for the less literate to comprehend to what they may be consenting. In addition, it is not clear that the routine collection and/or potential research use of CDRs is clearly disclosed in the policies of the major cell phone providers in SSA. In addition, awareness of research using CDRs among typical cell phone users in SSA is likely to be extremely low. For these reasons, it would not be uncharitable to mark this as an unconsented use of cell phone derived location data. The approach can be contrasted with other mobility research designs where participants are asked to explicitly agree to the collection and use of their data, such as the study by De Gruchy et al.^20^, which piloted the use of WhatsApp for the administration of surveys and collection of location data.

**Figure 1:**
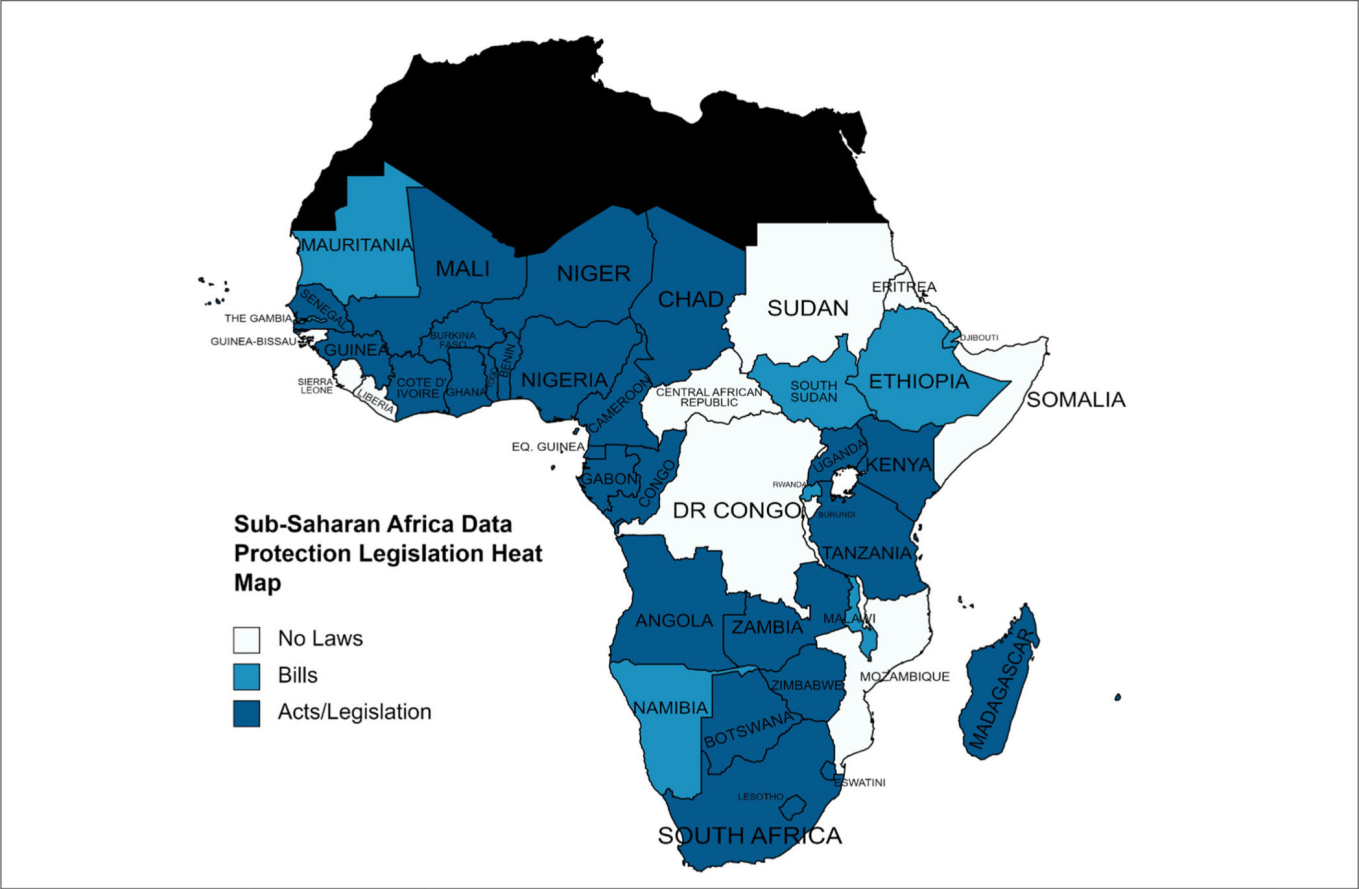
This map indicates data protection acts and legislation that govern the collection and use of personal data, which identifies living individuals directly or indirectly. Data protection bills are proposed laws or draft versions of laws or Acts under discussion and have not yet been passed by Parliament.

Arguably, the unconsented use of CDRs can be ethically acceptable if the data are fully anonymised, i.e. if the privacy dimension of autonomy is absolutely protected by rendering the reidentification of data sources impossible by all parties. Full anonymity is to be contrasted with deidentification (the removal of a person’s identifying characteristics, such as date of birth, from a data set) and pseudoanonymisation (where personal identifiers are replaced by pseudonyms or codes and cannot be attributed to a specific person without the aid of additional information).^21^ The latter imply that measures have been taken to render identification difficult, but not impossible. If the data are fully anonymised and reported in the aggregate, a balance can be struck between the potential social benefits of mobility research and the right to privacy. Such a balance is ethically ideal, but fragile, as the potential for reidentification needs to be continuously revisited as data sets are merged and algorithms become more sophisticated.^22^ Mobility data, as such, carries a risk of reidentification because movement patterns, even those of deidentified individuals, can reveal personal and possibly sensitive information.^23^ Repeated and frequent trajectories between one location (likely a person’s home or work), even of deidentified individuals, can reveal personal and possibly sensitive information, unless special care has been taken to ‘coarsen’ or aggregate spatial data. Deanonymisation via movement tracking can also pose threats to group privacy. Tracking the movements of displaced groups during humanitarian crises can be important for the provision of care and support, but the same technology can also be used, for example, by authoritarian regimes to track the activities of opposition groups. Even in countries like Ghana, which has a *Data Protection Act (Act 2012)*, there is a long list of exemptions – public order, public safety, public morality, national security or public interest – that enables a government to encroach on the rights of citizens.^24^ It should be noted that recent mobility research and big data capacity-building efforts in some African countries during the COVID-19 pandemic have established or consolidated close public–private partnerships between research institutions, national governments and telecommunications companies. Initiatives in Malawi^25^, the Democratic Republic of Congo^26^ and The Gambia^27^ aim to create a ‘pipeline’ of cell phone data for public health purposes, from telecommunications companies to ministries of health. How privacy is protected in these and other African pipelines will depend on the existence, nature and enforcement of national data legislation. Figure 1 provides a general overview of the status of data protection legislation in SSA as of April 2023. Enhancing the visibility of groups by governments through mobility data has ethical risks in the SSA context, particularly in relation to politicised ethnic and other divisions.^28^

### Bias and representativeness

The use of reliable scientific methods is a basic ethical requirement of health research.^29^ Unreliable methods risk producing invalid data, wasting research resources, and potentially harming individuals and communities if the data inform health policy. Like other types of research, mobility research faces challenges in regard to bias, accuracy and representativeness. In the SSA context, concerns have been raised about how to interpret cell phone derived mobility patterns when, for instance, individuals own and use more than one phone, or use different SIM cards in one phone, or when phones are shared by family members or friends.^30^ In their study in Uganda, Milusheva et al.^31^ noted the potential for bias in mobility data based on how phones are used: Ugandans (particularly those who are struggling financially) often prepay for phone services and use them intermittently. It is common for some phone users to switch off their GPS to conserve their cell phone’s battery, or they may be unable to keep their phones on due to the frequent power cuts that are common in many SSA countries. The result is that mobility data may disproportionately represent the movements of those who are male, relatively wealthy and largely urban, and therefore invite misleading inferences.^31^ For example, smartphone-derived data may suggest decreased mobility due to COVID-19 lockdown policies, but the decrease will be less than estimated if the data set does not include or account for the movements of those without smartphones and without the possibility to work from home.^32^ A study by Wesolowski et al.^33^ linked CDR and socio-economic survey data in Kenya to correct for cell phone ownership bias and attempted to produce a more representative estimate of mobility patterns. Contextualising phone-derived mobility data sets by comparing them with other data sources, and the use of complex filtering techniques are approaches to detect and reduce bias in mobility data sets that will be important considering the behaviour and circumstances of cell phone users in SSA.^34^

‘Mobility justice’ refers to how differences in class, gender, race, ethnicity, nationality, sexual identity and physical ability, as well as the built environment, social practices and public policies, influence human movement.^35^ The capacity and means to move about are not equitably distributed, and in SSA, mobility patterns commonly are shaped by class, gender and racial colonial histories and their legacies in the present. Nyamai and Schramm^36^ document how the colonial concentration of services and opportunities in Nairobi’s Central Business District and the prioritisation of public infrastructure for use by private vehicles compel the majority of citizens to travel long distances by (often inadequate) public transport or on foot. One could speak here of ‘thin’ and ‘thick’ conceptions of mobility. According to a thin conception, mobility is simply the movement of bodies through space and time as tracked by mobile devices. Under a thick conception, patterns of movement (or the lack thereof) are the result of a complex network of social determinants. This raises the question whether mobility research can or should engage with thick conceptions of mobility when interpreting patterns of movement and the justice-related considerations attached to them. For example, Deng and Wang^35^ found persistent disparities in the representativeness of movement data collected by social media and from cell phones in Texas and Louisiana during Hurricane Harvey, i.e. the data best reflected the movements of those in majority-white and non-poor neighbourhoods.

The precision of movement data also significantly differed by neighbourhood, which (like representativeness) is likely to influence how resources are allocated during natural disasters.^37^ They concluded that those collecting and interpreting mobility data should be aware of how minorities and low-income communities may become less visible and therefore less likely to receive assistance. In short, to avoid entrenching existing inequalities, mobility research needs to incorporate social justice considerations.

### Community awareness, engagement and trust

Although COVID-19 has accelerated public health research using cell phone data, studies of public awareness, knowledge or attitudes towards the use of such data for public health purposes are conspicuous by their absence. Jones et al.^38^ reviewed published research on the challenges and opportunities of using CDRs in health research, and could not find any literature on public perceptions of using such records. More recently, a scoping review by Sekandi et al.^39^ on ethical, legal and socio-cultural issues in the use of CDRs for public health in the East African region found no published research on public views on this topic. Relatedly, there appears to have been little or no community engagement in health-related mobility research using CDRs in Africa or elsewhere.^39^Revealingly, a recent set of guiding principles to maintain public trust in the use of mobile operator data proposed by a group of statisticians, data analytics specialists and academics does not appear to regard community input as important to the maintenance of trust.^40^ They appear to assume that as long as key stakeholders (i.e. government agencies, data analysis organisations and mobile device operators) follow principles of necessity and proportionality, professional independence, privacy protection, quality control and international comparability, community engagement is unnecessary. On this view, public trust simply follows from stakeholder trustworthiness:

In all, explicitly addressing the five principles in the preparation of a project should give confidence to the statistical agency and its partners, that enough care has been exercised in the set up and implementation of the project, and should convey trust to public and government in the use of mobile operator data for policy purposes.^40(p.e24−1–e24−21)^

The neglect of community engagement in health-related mobility research using cell phone data is problematic for a number of reasons. First, at least some degree of community engagement is increasingly expected (if not demanded) by regulators and ethics committees as a basic requirement of the responsible conduct of research.^41^ Raising awareness that mobility research using CDRs is taking place would be a bare minimum level of engagement. Second, using data from communities without their awareness or input, even if following professional standards, is a potential source of mistrust, suspicion and misinformation. Community acceptance of basically unconsented data collection for public health purposes cannot be assumed. A study by Garrett and Young^42^ on patient views on the use of digital data for public health surveillance suggests that the public may be significantly less comfortable about the collection and use of location data than with data from social media accounts or electronic health records. Third, not involving the public is a lost opportunity for improving the quality of mobility data by addressing possible gaps between how movements are represented and how (and perhaps why) people are actually moving. Lastly, community engagement could help identify risks to community members posed by mobility research that may be invisible to data scientists, particularly those who have little familiarity with the societies they are studying.^43^

### Function creep and accountability

‘Function creep’ refers to the phenomenon of a technology being used for something other than its originally intended purpose. Drivers’ licences, originally meant to promote traffic safety, gradually took on the role of authorised personal identification. The use of CDRs for public health promotion is itself a form of function creep, although ethical concerns about function creep typically are about when the new function of the technology is less benign than its original purpose.

As some in technology studies have observed, the ‘creep’ in function creep often takes the form of a gradual expansion from a context of care to a context of control.^44^ Here, there is a concern that techniques developed in cell phone data-driven mobility research to promote public health in LMICs will end up being used for questionable surveillance, commercial or political purposes. For example, some experts suggest that uses of cell phone data to help tackle humanitarian crises and infectious diseases in LMICs are very likely to be repurposed to predict, track and prevent unwanted migration.^45,46^ Foreign involvement in the African communication technology sector, particularly that of the Chinese government, has also raised questions about function creep and accountability.^47^ Chinese companies like Huawei and the Transsion Group have invested heavily in the cell phone infrastructure of SSA. While the provision of loans, equipment and training initiatives has stimulated needed growth in this sector, the Chinese government and its corporate track record of the digital surveillance of its own citizens raises concern that mobile data from Africans may be transmitted abroad and that African governments are being assisted to use mobile technology to increase social control over their citizens.^48^ The Chinese government and Chinese companies are not the sole focus of these concerns: the actions of Western governments and companies have also increased the risk for ‘digital authoritarianism’ in Africa.^49^

As more and more digital phone data are being collected, analysed and shared, potentially harmful manifestations of function creep are likely inevitable. A question then is: who is accountable for minimising the risks raised by function creep in regard to cell phone derived mobility data in SSA? COVID-19 and the rise of data digitisation have stimulated the further development of legal and regulatory frameworks for data protection and privacy, although currently the result is a patchwork, with some African countries having few or no relevant policies, while others have extensive governance frameworks.^50^ However, the use of CDR data for public health is relatively new in SSA, and it is unclear how well this particular form of data is covered, even by the most developed policies. In the meantime, ad hoc agreements continue to be reached between cell phone companies, government agencies, researchers and data analytics organisations in their collaborative projects, with each stakeholder answering to their own internal regulatory regime. At present, there do not appear to be overarching governance structures within SSA countries to minimise the risk of cell phone derived mobility data being used for ethically questionable purposes. Developing such structures appears to be a matter of urgency, as African governments leverage cell phone technology to take actions that can significantly impact citizens, while bypassing public debate. In Ghana, for example, the government has made a digital identification card compulsory, while requiring SIM cards from all cell phones to be linked to the digital ID.^51^ At the same time, the government has introduced a 1.5% levy on all financial transactions conducted by cell phone, which will disproportionately impact Ghanaians of lower socio-economic status.^52^ This suggests that, while national governments are the ‘natural’ authorities responsible for minimising negative forms of function creep, governments themselves need to be held publicly accountable for how they make use of cell phone data and why.

### Stakeholder relationships and power dynamics

Ethical issues regarding research data governance in Africa have been raised for many years, particularly in response to large-scale genomic research initiatives, such as H3Africa.^53^ However, what sets mobility data apart is that researchers do not collect the primary data themselves: they depend on commercial entities, i.e. cell phone operators, from whom they gain CDR data through data use agreements. The ethical implications of this relationship in the SSA context are relatively unexplored, but, clearly, cell phone operators become key public health stakeholders in an arrangement where researchers and governments come to depend on them for public health related data. This is part of a larger global issue: digital technology companies are increasingly playing a central and profit-seeking role in public sectors traditionally governed by states (such as emergency response, national security, education and law enforcement), but without being subject to the accountability, transparency or legitimacy of state agencies.^54^ Our main interest here, however, is where mobility researchers and their research institutions are situated within this new landscape. Although not seeking to make a profit, they use CDR data obtained by commercial entities from individuals with minimally informed and questionably voluntary consent. Even if the mobility data are anonymised, the original commercial consent standards under which the data were obtained falls significantly below traditional research standards for consent, and, given the vast numbers involved, reconsent is out of the question. This arrangement also complicates the ethical review of research: even with ‘broad consent’ for the use of biological samples, research participants at least know their data are being collected and will be used in future studies. Cell phone users, whose data are collected passively, know far less about the potential use of their data in mobility (and other) research. Under what conditions should research ethics committees approve the use of such data for research purposes? Further, to the extent that mobility analysis informs public policy, mobility researchers and their institutions in this way entrench and normalise the influence of commercial technology firms on the public domain, despite their primary motivation being public health promotion.

In short, the stakeholder relationships in this relatively new field in LMICs risk being marked by dominance, dependence, a lack of transparency and disempowerment along a number of lines: the power held by cell phone companies with vast amounts of citizens’ mobility data; the data dependency of governments and researchers on the companies; the lack of control by individuals and communities over the collection, sharing and use of the data collected from them; and the unwillingness or inability of governments to hold companies accountable in ways commensurate with their growing public influence. This risk is particularly significant in SSA countries, where national governments in resource-limited settings with weak health infrastructures may be highly vulnerable to coming under the sway of powerful transnational corporations. In addition, some African governments have a poor track record in regard to public accountability. The commercially mediated use of big data by political authorities could further widen the rift between government and the governed.

### Translation of mobility analyses into health policy

Public health research is conducted on the assumption that its findings can be used to improve health by informing relevant policies and practices. Leaving to one side the issues of data representativeness and accuracy, a central question is what health policymakers should do with cell phone derived mobility analyses. As with other emerging and exciting information technologies, there is a risk of this approach being regarded as inherently superior to other ways of generating evidence or to other considerations that are important to health policy decision-making. This can lead to an overly technology-driven policy approach, such as can be seen in critiques of how big data have been utilised in the development of ‘smart cities’. As Kitchin^55^ argues, heavy reliance of urban policy on ‘real time’ big data analytics, combined with a neglect of ethical considerations and the lived experience of city dwellers, threatens to make cities *less* inhabitable.

Current debates about evidence-based policy indicate that, while having good evidence is crucially important, health policy is always, to some extent, underdetermined by empirical evidence.^56^ For example, a number of studies have been conducted comparing the implementation of COVID-19 lockdown policies with mobility as derived from cell phone location data in different settings over specific time periods.^57^ Many mobility studies suggest that stay-at-home policies slowed the spread of COVID-19 at the beginning of the pandemic by inhibiting movement and association, but were less effective as time went on. What does such mobility information imply for future pandemic policymaking? Different policy directions are possible. One could argue that stay-at-home orders have a limited effect over time, and such policies should be used sparingly in the future, particularly in the light of the negative social effects of the large-scale inhibition of movement, i.e. impacts on mental health and child development. One could also argue, with the same data, that stay-at-home orders had a very significant effect on viral spread, and such policies should be more strictly enforced and should be enforced for longer durations in the future. Whatever path is taken will be an evidence-informed result of political, social, legal and ethical deliberations. The same holds for mathematical models, which make use of CDR data to capture (for example) the extent of a disease outbreak or predict the effects of different health policy options.^58^ Mobility data alone cannot answer health policy questions that are essentially normative in nature, and which incorporate issues of fairness, as well as those of economics and viral control.

In LMICs, including those in SSA, health policymaking is often ad hoc and fragmented in many chronically under-resourced ministries of health.^59^ Human and infrastructural resources will need to be significantly strengthened before many public health systems are in a position to meaningfully utilise the digital data that mobility researchers are gathering. To translate mobility data into valuable policy information, mobility data experts have underlined the importance of developing standardised procedures and mechanisms that are responsive to legal and ethical considerations.^16^ Even in high-income countries such as the USA, the massive amounts of digital data collected during the COVID-19 pandemic had little public health impact due to enormous gaps in the translational pipeline.^60^ A situation marked by scarcity of local data science expertise, weak regulatory regimes, little to no community engagement and public health systems not yet prepared to absorb digital data collected from Africans is bound to raise questions about what will be done with the data that continue to be collected, what the real benefits are, and who stands most to gain.

## Conclusion

Improving our understanding of human behaviour is vitally important for efforts to improve public health. Insights from cell phone derived mobility data could be beneficial in many contexts, including humanitarian disasters, infectious disease outbreaks and responses to climate change. Considering persistent population health challenges and the sharp growth in cell phone use in SSA, it is understandable that public health researchers, organisations and policymakers are excited about the potential beneficial applications of mobility data. In this paper, we identified key challenges to be taken into account in the collection, sharing, management and use of mobility data in this setting. Moving forward, greater attention will need to be paid to the governance environments in respective SSA countries in regard to this specific type of data, and, in particular, how mobility data are shared between private mobile operators, researchers, national governments and other third parties. It is important to have accurate local knowledge about circumstances where seemingly innocuous information about human movement can become ethically sensitive, such as regions with territorial disputes, jurisdictions that criminalise sexual minorities, or places where religious groups are persecuted. To date, very little social science research has been conducted in SSA about the potential risks of social harm related to mobility data. Social science research on community attitudes about mobility data use is also in its infancy; a recent qualitative study in South Africa suggests that only a minority of those interviewed were concerned about the use of their location data, but also noted that the majority did not really know how that data were being used.^61^ Relatedly, increasing the engagement of communities and civil society organisations will be important for the ethical use of mobility data in public health research and policy, especially in efforts to hold both private companies and governments accountable. Local research ethics committees can also contribute to accountability efforts, although their effectiveness will likely depend on increasing knowledge of big data research among committee members.^62,63^ Data ethics tools have been developed in a number of countries (such as The Data Ethics Canvas of the Open Data Institute, The Box by AI Ethics Lab, and the Data Ethics Decision Aid of Utrecht University) that could be of some use for research ethics committees in SSA. In short, there are some identifiable challenges, but much is unknown, and much is left to be done in regard to the ethical use of cell phone derived mobility data in the SSA context.

## Data Availability

☐ Open data set ☐ All data included ☐ On request from author(s) ☐ Not available ☒ Not applicable

## Significance:

- Mobility data derived from cell phones are being increasingly used for health research and public health purposes in sub-Saharan Africa, with minimal individual consent and largely without public awareness.
- While such data can have significant potential public health benefits, risks and concerns related to their collection and use in sub-Saharan African contexts have not been widely discussed.
- Innovative community engagement initiatives, which are appropriate and responsive to sub-Saharan African contexts, need to be developed to address ethical challenge areas and help warrant public trust in mobility research.
